# IGF2BP3 regulates the expression of RRM2 and promotes the progression of rheumatoid arthritis via RRM2/Akt/MMP-9 pathway

**DOI:** 10.1371/journal.pone.0303593

**Published:** 2024-05-31

**Authors:** Zhaonan Ban, Zhengjiang Li, Shuxing Xing, Yongjie Ye

**Affiliations:** Department of Orthopedics, Chengdu Fifth People’s Hospital, Chengdu, Sichuan, 611130, China; Ruijin Hospital, Shanghai Jiao Tong University School of Medicine, CHINA

## Abstract

**Background:**

Rheumatoid arthritis (RA) is a common inflammatory and autoimmune disease. Ribonucleotide Reductase Regulatory Subunit M2 (RRM2) is a crucial and a rate-limiting enzyme responsible for deoxynucleotide triphosphate(dNTP) production. We have found a high expression level of RRM2 in patients with RA, but the molecular mechanism of its action remains unclear.

**Methods:**

We analyzed the expression of hub genes in RA using GSE77298 datasets downloaded from Gene Expression Omnibus database. RRM2 and insulin-like growth factor-2 messenger ribonucleic acid (mRNA)-binding protein 3 (IGF2BP3) gene knockdown was achieved by infection with lentiviruses. The expression of RRM2, IGF2BP3, matrix metalloproteinase (MMP)-1, and MMP-9 were detected via western blotting assay. Cell viability was detected via 3-(4,5-dimethylthiazol-2-yl)-2,5-diphenyltetrazolium bromide (MTT) assay. MeRIP-qRT-PCR was performed to test the interaction of IGF2BP3 and RRM2 mRNA via m6A modification. Cell proliferation was determined by clone formation assay. Migration and invasion assays were performed using transwell Boyden chamber.

**Results:**

RRM2 and IGF2BP3 were highly expressed in clinical specimens and tumor necrosis factor alpha (TNF-α) and interleukin (IL)-1β-stimulated synovial cells. RRM2 and IGF2BP3 knockdown inhibited the proliferation, migration, and invasion of MH7A cells. The inhibitory effects of IGF2BP3 knockdown were effectively reversed by simultaneously overexpressing RRM2 in MH7A cells. By analyzing N6-methyladenosine (m6A)2Target database, five m6A regulatory target binding sites for IGF2BP3 were identified in RRM2 mRNA, suggesting a direct relationship between IGF2BP3 and RRM2 mRNA. Additionally, in RRM2 small hairpin (sh)RNA lentivirus-infected cells, the levels of phosphorylated Akt and MMP-9 were significantly decreased compared with control shRNA lentivirus-infected cells.

**Conclusion:**

The present study demonstrated that RRM2 promoted the Akt phosphorylation leading to high expression of MMP-9 to promote the migration and invasive capacities of MH7A cells. Overall, IGF2BP promotes the expression of RRM2, and regulates the migration and invasion of MH7A cells via Akt/MMP-9 pathway to promote RA progression.

## Introduction

Rheumatoid arthritis (RA) is a common autoimmune disease characterized by persistent synovitis and cartilage destruction [1, 2]. In the course of RA progression, the activated synoviocytes exhibit proliferative and invasive features, which lead to bone erosion and the destruction of cartilage [3]. Patients often experience persistent joint pain, swelling, and stiffness, and serious cases may lead to cardiovascular, lung, bone, and other complications [4]. It has been reported that RA has a high incidence rate in China and worldwide and can occur at any age. At present, the main drugs commonly used for RA treatment include non-steroidal anti-inflammatory drugs, glucocorticoids, anti-rheumatic drugs, biological products, and traditional Chinese herbal medicine [5–7]. Because the pathogenesis of RA is still unclear, its elucidation has important guiding significance for clinical treatment and research and development of new drugs.

Ribonucleotide reductase (RNR) is responsible for deoxyribonucleic acid (DNA) synthesis and repair via regulating deoxyribonucleotide triphosphate synthesis. RNR consists of two subunits: RNR large subunit M1 (RRM1) and RNR small subunit M2 (RRM2). RRM2, a critical protein for DNA synthesis and repair, catalyzes the biosynthesis of deoxyribonucleotides from the corresponding ribonucleotides [8, 9]. We have found that RRM2 was an upregulated hub gene that promoted the progression of RA [10]. Wu et al. reported that RRM2 was highly expressed in RA and served as a novel biomarker for the diagnosis of RA using peripheral blood mononuclear cells (PBMCs) [11]. Another group found that RRM2 was correlated with ferroptosis in RA progression [12]. RRM2 short interfering ribonucleic acid (RNA) delivery effectively decreased the proliferation of RA fibroblast-like synoviocytes (RA-FLS) and inhibited the secretion of tumor necrosis factor (TNF)-α and interleukin (IL)-6 [13]. However, no further mechanisms in RA progression were investigated till now. We have found that several micro-RNAs (miRs), including miR-106A-5p, miR-20a-5p, and miR-17-5p negatively regulated the RRM2 expression during RA progression[10]. In patients with lung adenocarcinoma (LUAD), low RRM2 was associated with a better prognosis because miR-202-3p inhibited the proliferation and metastasis of lung cancer cells by targeting RRM2 [14]. Additionally, circ_0039908/miR-let-7c/RRM2 axis was found to promote the proliferation and migration of LUAD cells [15].

In mouse embryo fibroblasts, RRM2 expression level was increased through beta-catenin related pathway [16]. Moreover, it has been found that RRM2 regulated the proliferation and repressed apoptosis by activating phosphoinositide 3-kinases (PI3K)/Akt pathway in retinoblastoma cells [17] and non-small cell lung cancer (NSCLC) cells [18]. In the present study, we aimed to further explore how RRM2 regulates RA progression.

N6-methyladenosine (m6A) modification has been reported to regulate various human diseases, such as different human cancers, cardiovascular diseases, osteoarthritis, and RA [19–22]. The m6A modification process was regulated by the regulatory proteins, including m6A writers, erasers, and readers [23–25]. The m6A modifications contribute to messenger RNA (mRNA) degradation, translation, or stabilization, which are reversible and normally seen in eukaryotic mRNAs. The m6A modifications exhibit different distributions among tissue types and stress conditions suggesting unique m6A modification patterns dependent on different cell type and cell state [26]. To date, only few studies focused on m6A modification during RA progression. The results of transcriptome-wide hi-throughput m6A sequencing in RA-FLS showed that the m6A methylation-related genes, including Wilms tumor 1 associated protein, receptor interacting serine/threonine kinase 2, Janus kinase 3, and TNF receptor superfamily member 10a, were associated with the occurrence and development of RA [27]. Geng et al. reported that insulin-like growth factor-2 mRNA-binding protein 3 (IGF2BP3) and YTHDC2 were differentially expressed in patients with RA and controls, which had a high diagnostic value [19]. Moreover, it has been found that methyltransferase 3 and YTHDF2 cooperatively modify and degrade the expression of peroxisome proliferator activated receptor gamma coactivator 1 alpha, cytochrome c, somatic, nicotinamide adenine dinucleotide (NAD)^+^ hydrogen: ubiquinone oxidoreductase subunit C2, and OCR in inflammation-associated diseases, including RA [28].

It has been detected previously that RRM2 was highly expressed in RA, suggesting that RRM2 promoted the progression of RA. In the present study, we further explored the possible m6A regulatory mechanism of RRM2 in RA progression. The results of m6A2Target database analysis showed that RRM2 mRNA had five possible IGF2BP3-binding sites. IGF2BP3 is the well-known m6A reader that regulates m6A modification in disease progression. This study aimed to elucidate whether and how RRM2 was regulated by IGF2BP3 in RA progression. Considering the over-expression of RRM2 in RA progression, it could be used as an effective therapeutic target. Moreover, several inhibitors of RRM2 might be used for clinical RA treatment. Thus, IGF2BP3 possesses potential therapeutic implications of targeting RRM2, and further clarification of the RRM2/m6A modification related mechanism in RA progression is required.

## Material and methods

### Identification of the hub genes in patients with RA

The GSE77298, GSE55235 and GSE55247 datasets were downloaded from the Gene Expression Omnibus (http://www.ncbi.nlm.nih.gov/geo). We compared the differentially expressed genes (DEGs) in the GSE77298, GSE55235 and GSE55247 datasets using Xiantao platform (https://www.xiantao.love/,). The cut-off criteria were |log2 (FC)| > 1 and adjusted p < 0.05, and DEGs of GSE77298 were shown using a volcano map (S1 Table). The top 10 hub genes were clarified by performing PPI network analysis of DEGs in STRING database and analyzing it with Cytoscape software as described [10]. The expression values of Top10 genes were represented as mean values ± SD.

### Cell Lines, clinical specimens, and reagents

The human RA-FLS cell line MH7A cells were obtained from JENNIO Biological Technology (Guangzhou, China). The cells were cultured in Dulbecco’s modified eagle medium (DMEM) supplemented with 10% fetal bovine serum (FBS) at 37°C in 5% CO_2_ atmosphere. MH7A cells were incubated with TNF-α (10 ng/mL; Cat. no. 14832962; Thermo Fisher Scientific Inc.) or IL-1β (10 mg/L; Cat. no. SRP3083; SigmaAldrich Corp., St. Louis, MO, USA) for 24 h to simulate RA cells. Four pairs of intraoperative synovial specimens were collected from the Orthopedics Department at Chengdu Fifth People’s Hospital from June 2019 to March 2021. The clinical samples were obtained from patients with RA who underwent total knee arthroplasty and control patients with ligament injury caused by exercise or mechanical force. The age range of patients was 37.2–52.6 years old and the median age was 46.8. This study proceeded according to Declaration of Helsinki (2013) and was approved by the Ethics Committee at Chengdu Fifth People’s Hospital (approval no. 2019B-0127; Chengdu, China). All patients or their parent/legal guardian provided written informed consent for participation.

### Lentivirus packaging and gene expression

RRM2 Lentiviral copy DNA Open Reading Frame Clone, Human, C-GFPSpark® tag (Cat: HG18284-ACGLN) and pLV-C-GFPSpark lentivirus control plasmid (Cat: LVCV-35) were purchased from SinoBiological corporation (Beijing, China). Lentiviral vectors encoding RRM2 were transfected with psPAX2 packaging and pMD2.G envelope plasmid to HEK293T cells (Cat. No. CL-0005, Procell, Wuhan, Hubei, China) for 2 d using Lipofectamine 3000 (Invitrogen). Moreover, MH7A cells were infected with viral supernatants in the presence of 8 μg/mL polybrene.

### RRM2 small hairpin (sh)RNA and IGF2BP3 shRNA lentiviral particles transduction

IGF2BP3 shRNA (h) lentiviral particles (cat.No. sc-60846-V) and control shRNA lentiviral particles (Cat. No. sc-108080) were purchased from Santa Cruze. RRM2 human shRNA lentiviral particles (Locus ID 6241, Cat.No: TL309700V) and control lentiviral particles (Cat.No.: TR30021V) were purchased from Origene. The lentivirus infection was performed according to the manufacturer’s protocol. Briefly, MH7A cells (4x10^4^ cells/per well of 24-well plate) were plated and incubated overnight at 37 ˚C in 5% CO_2_. The lentiviral particles were added into the culture medium at a multiplicity of infection (MOI) of 50 at a total volume of 500 μL supplemented with 8 μg/mL Polybrene® (cat. No. sc-134220, Santa Cruze). After 24 h, the fresh culture medium without Polybrene was added. Stable clones expressing RRM2 shRNA or IGF2BP3 shRNA or control shRNA were screened according to kit protocols.

### 3-(4,5-dimethylthiazol-2-yl)-2,5-diphenyltetrazolium bromide (MTT) assay

MTT assay was performed to test cell viability as described previously. Briefly, MH7A cells were infected with RRM2, shRNA, or control shRNA lentiviruses for 24 h, 48 h, and 72 h, respectively. In the other experiment, the cells were infected with IGF2BP3 shRNA lentivirus or control shRNA lentivirus for the indicated time. Additionally, the MH7A cells were infected with IGF2BP3 shRNA lentivirus or control shRNA lentivirus combined with RRM2 overexpression lentivirus or control lentivirus, respectively, for the indicated time. The optical absorbance of the samples was measured at 490 nm on a scanning multi-well spectrophotometer. Each treatment was administered in triplicate.

### Methylated RNA immunoprecipitation qRT-PCR (MeRIP-qRT-PCR)

MeRIP was performed as described below. Briefly, MH7A cells infected with IGF2BP3 shRNA or control shRNA lentiviruses were incubated with TNF-α or IL-1β for 24 h. RNA was extracted and purified to deplete the ribosomal RNA and avoid DNA contamination. Protein A/G magnetic beads conjugated with anti-m6A antibody (ab151230, abcam, USA) or mouse IgG were incubated with cell lysates supplemented with RNase inhibitor at 4°C overnight. M6A-modifed mRNAs were eluted from the beads with elution buffer and then purification was carried out for further analysis by RT-qPCR. Relative enrichment was normalized to the input. The relative mRNA expression levels were determined using the 2−ΔΔCt method. The primer sequences used were as follows: RRM2-Forward: 5’-CTGGCTCAAGAAACGAGGACTG-3′, and RRM2-Reverse: 5’-CTCTCCTCCGATGGTTTGTGTAC-3′; GAPDH-Forward: 5’-CAGCGACACCCACTCCTC-3′ and GAPDH-Reverse: 5’-TGAGGTCCACCACCCTGT-3′.

### Western blotting assay

After infection with lentiviruses for 48 h, MH7A cells were collected and lysed with radioimmunoprecipitation assay lysis buffer. The total protein concentration was determined using bicinchoninic acid kit. Each lane was loaded with the total protein at 20 μg/well. 10% sodium dodecyl sulfate–polyacrylamide gel electrophoresis (SDS-PAGE) was used to separate the total proteins. Following SDS-PAGE and blotting, the membranes were incubated with primary antibodies, including anti-RRM2 rabbit polyclonal antibody (Cat.No. HPA056994) purchased from Amylet Scientific (Wuhan, China). IGF2BP3 Polyclonal antibody (Cat no: 14642-1-AP) was purchased from Proteintech (Rosemont, IL, USA). Rabbit anti-Akt Antibody #9272 and Phospho-Akt (Ser473) (587F11) mouse mAb #4051 were purchased from Cell Signaling Technology (MA, USA). Anti-MMP9 (ab38898) and anti-MMP1 antibodies [EP1247Y] (ab52631) were purchased from Abcam. Rabbit anti-Actin Antibody (A2066) was purchased from Sigma.

### Clone formation assay

Approximately 1,000 cells were plated at 12-well plates in DMEM supplemented with 10% FBS for the colony formation assay. After 2 weeks of incubation, the surviving colonies were fixed, stained with 0.5% crystal violet, imaged, and counted.

### Migration and invasion assays

MH7A cells were infected with RRM2 shRNA /control shRNA lentivirus, or IGF2BP3 shRNA/control shRNA lentiviruses, and the stable cell lines were selected as described above. Migration and invasion assays were performed using 24-well plates with Transwell membrane filter inserts (cat. no. 3422; Corning Costar, Inc.) without or with precoated diluted Matrigel (cat. no. 354248; BD Biosciences). Briefly, 1x104 cells/well in serum-free culture medium were seeded into the upper chambers of a 24-well Transwell chamber (pore size, 8 μm). After culturing for 24 and 48 h, the cells on the lower surface of the filter were fixed with 10% formalin solution for 30 min and stained with 0.1% crystal violet for 30 min.

### Statistical analysis

Every experiment was performed in triplicate and repeated at least twice independently. The results were presented as the mean ± standard deviation. One-way analysis of variation followed by Tukey’s post hoc test was used for comparisons between >2 groups. p<0.05 was considered to indicate a statistically significant difference.

## Results

### Top 10 hub genes associated with RA validated in GSE77298, GSE55235 and GSE55247 datasets

**Fig 1:**
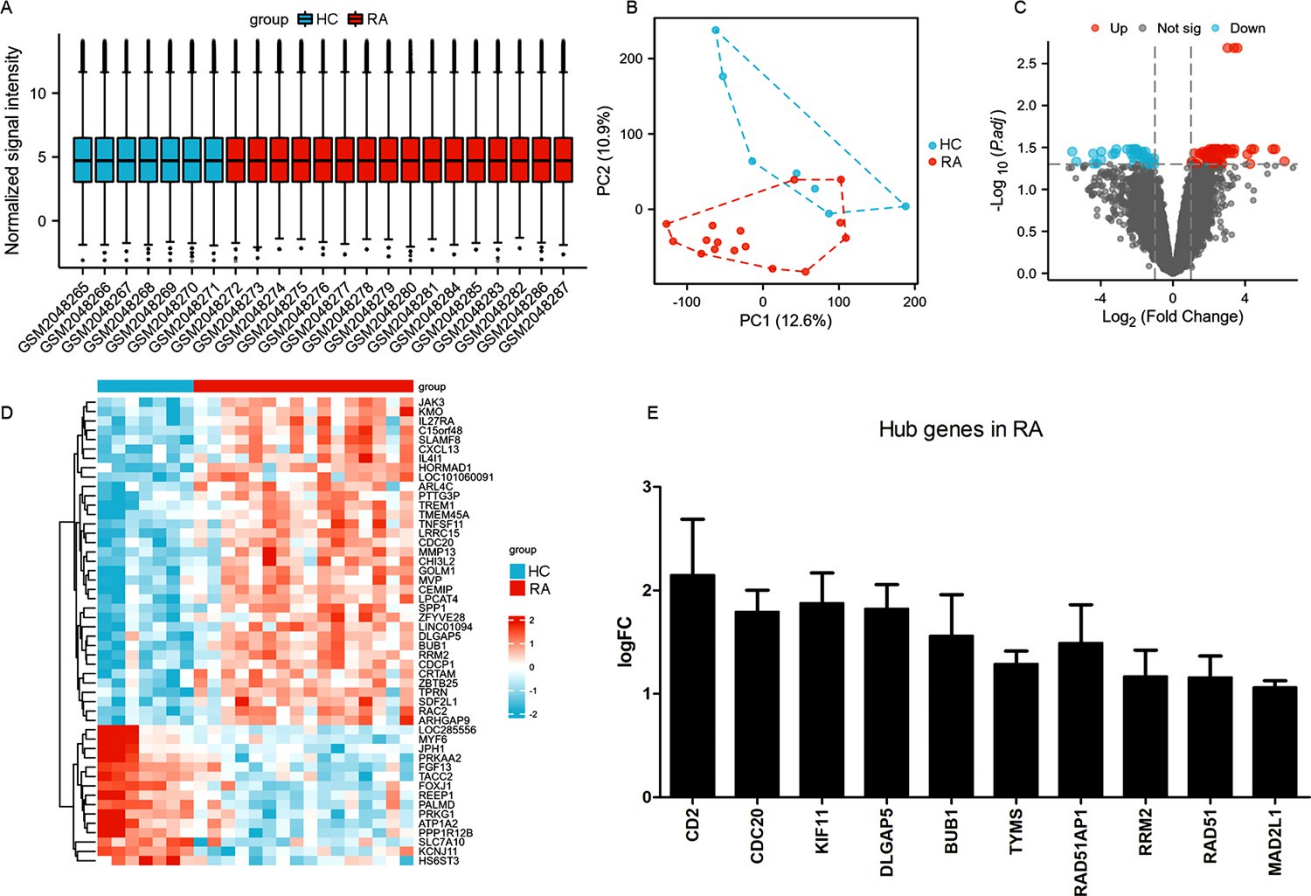
Top 10 hub genes associated with RA validated in GSE77298. A. The data were normalized and presented by box chart. B. Principal component analysis (PCA) for dimension reduction. C. Volcano plots performed for differential analysis. D. Heat map performed to show the differential expression genes of GSE77298. E. The top 10 hub genes from patients with RA and controls from GSE77298, GSE55235 and GSE55247 datasets shown in histogram.

Previously, PPI network was constructed based on 174 overlapping DEGs associated with RA in GSE55235 and GSE55457 using STRING, and Top 20 hub genes were identified by using Cytoscape as described [10]. In the present study, we selected GSE77298 as the validation dataset. As shown in Fig 1A & 1B, the data were normalized and shown by box chart followed by PCA analysis. Next, the differentially expressed genes were also shown by volcano plots (Fig 1C). Then, we tested the Top 10 hub genes identified as related to RA pathogenesis in the GSE77298, GSE55235 and GSE55247 datasets. We found that the expression of 10 hub genes, including BUB1, RAD51AP1, DLGAP5, kinesin family member 11(KIF11), MAD2L1, RAD51, thymidylate synthase (TYMS), RRM2, cell division cycle 20(CDC20), and cluster of differentiation 2(CD2), were all upregulated in patients with RA in the dataset GSE77298, GSE55235 and GSE55247 datasets (Fig 1D). Among them, RRM2 was one of the significantly upregulated genes in the progression of RA, but the molecular mechanism in the pathogenesis of RA was not clearly clarified [10]. Here, we aimed to explore the role and molecular mechanism of RRM2 in the pathogenesis of RA.

### RRM2 is upregulated in the synocytes of patients with RA and TNF-α and IL-1β-treated MA7H cells

**Fig 2:**
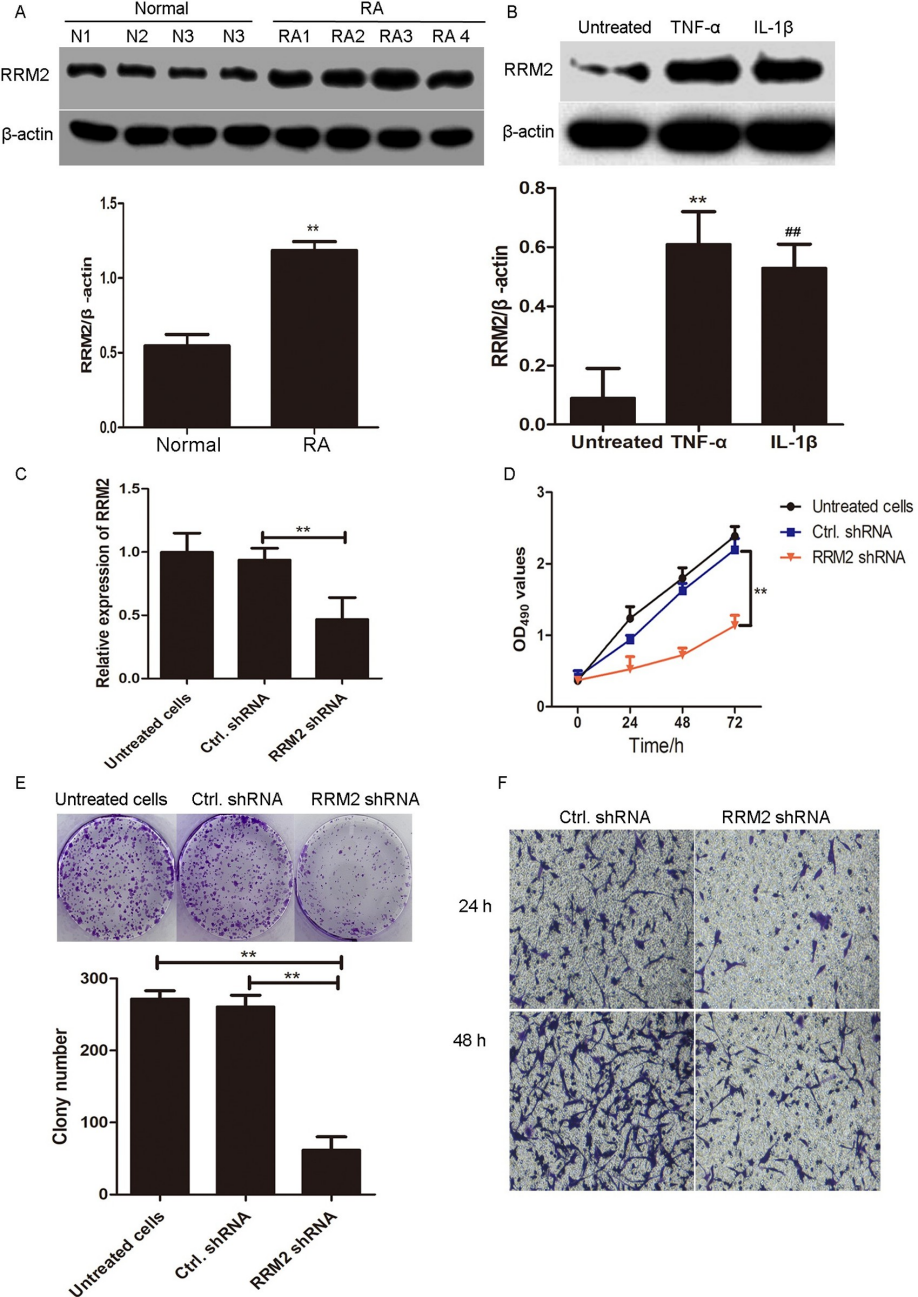
RRM2 knockdown inhibits the proliferation, migration, and invasion of RA-FLSs. A. The expression of RRM2 was detected via western blotting in synovial specimens from patients with RA and normal controls. The relative expression of RRM2 was shown in histogram. **p<0.01, compared to normal controls. B. MH7A cells were treated with TNF-α (10 ng/mL) and IL-1β (10 mg/L) for 24 h, and the expression of RRM2 was determined via western blotting. The relative expression of RRM2 was shown in histogram. **p<0.01, compared with untreated group. ##p<0.01, compared with untreated group. C. MH7A cells were treated with RRM2 shRNA lentivirus and lentiviral controls for 24 h. The relative expression of RRM2 was determined via western blotting and shown in histogram. **p<0.01, compared with control lentivirus group. D. MTT assay. MH7A cells were infected with RRM2 shRNA lentivirus and lentiviral controls for 24, 48, and 72 h, and cell viability was determined via MTT assay. **p<0.01. E. Clone formation assay. MH7A cells were infected with RRM2 shRNA and control shRNA lentivirus and cultured for 2 weeks. Colony formation assay was performed as described in Materials and Methods. The colony number was shown in histogram. **p<0.01, compared with control shRNA group. F. Migration and invasion assays. MH7A cells were infected with RRM2 shRNA and control shRNA lentiviruses and treated with 10 ng/mL of TNF-α. The migratory and invasive abilities were detected using transwell Boyden chamber coated without or with a Matrigel basement membrane matrix after 48 h. Original magnification ×100.

To clarify the role, as well as the molecular mechanism of RRM2 in the pathogenesis of RA, we tested the RRM2 expression in four pairs of intraoperative synovial specimens from patients with RA and control patients. As shown in Fig 2A, the expression of RRM2 was significantly increased in clinical synovial specimens from patients with RA compared to that from controls. Moreover, RA was accompanied relentlessly by inflammation; thus, we used MH7A cells being treated with TNF-α (10 ng/mL) or IL-1β (10 mg/L) for 24 h to imitate the RA-related inflammation. As shown in Fig 2B, the expression of RRM2 was significantly increased in TNF-α and IL-1β-treated MH7A cells.

### RRM2 knockdown inhibits the proliferation, migration, and invasion of MH7A cells

We interfered with the endogenous RRM2 expression by infection with RRM2 shRNA lentivirus, and quantitative (q) reverse transcription polymerase chain reaction (RT-PCR) results showed that the RRM2 expression was significantly downregulated in RRM2 shRNA lentivirus-infected MH7A cells compared to that of control shRNA lentivirus-infected cells (**p<0.01, Fig 2C). The cell viability of RRM2 shRNA lentivirus-infected MH7A cells was significantly decreased compared to that of control shRNA lentivirus-infected MH7A cells at 24 h, 48 h, and 72 h (**p<0.01, Fig 2D). Consistently, the number of cell clones in RRM2 shRNA lentivirus-infected MH7A cells was significantly decreased compared to that of control shRNA lentivirus-infected MH7A cells (Fig 2E). Furthermore, the migratory and invasive abilities of RRM2 shRNA lentivirus-infected MH7A cells were significantly decreased compared with those of control shRNA lentivirus-infected cells at 48 h (p<0.01) (Fig 2F). The results revealed that interference with RRM2 suppressed the proliferation, migration, and invasion of MH7A cells.

### RRM2 mRNA has five possible IGF2BP3 binding sites

We wanted to identify whether RRM2 expression was affected by m6A methylation in RA pathogenesis. First, the expression of key m6A regulatory genes was compared with expression data in GSE77298 (Table 1). The results showed that the expression of m6A reader IGF2BP3 was significantly increased in patients with RA. By analyzing m6A2Target database (http://m6a2target.canceromics.org), a comprehensive database for targets of m6A writers, erasers, and readers, we found that there were five binding sites of RRM2 in the second chromosome (Chr2), including chr2: 10130913–10130933; chr2: 10127121–10127143; chr2: 10131380–10131410; chr2: 10122859–10122884; chr2: 10129417–10129439, which might be the targets of IGF2BP3 (Table 2).

**Table 1:** The m6A regulatory genes in GSE77298.

ID	Gene symbol	LogFC	P value	WER
209265_s_at	METTL3	0.206461891	0.445949221	Writer
235552_at	METTL14	-0.1770083	0.43095833	Writer
229630_s_at	WTAP	0.54665922	0.04200092	Writer
219286_s_at	RBM15	-0.0635569	0.78261011	Writer
212402_at	ZC3H13	-0.0725995	0.69210086	Writer
209702_at	FTO	0.04105887	0.88424554	Eraser
234302_s_at	ALKBH5	-0.0449568	0.9001993	Eraser
221741_s_at	YTHDF1	0.13694406	0.45765919	Reader
217812_at	YTHDF2	0.24724809	0.19011141	Reader
221749_at	YTHDF3	-0.252945	0.20356208	Reader
212455_at	YTHDC1	-0.1878357	0.37305925	Reader
213077_at	YTHDC2	-0.2889565	0.1797285	Reader
236700_at	EIF3C	-0.2236649	0.5571242	Reader
205292_s_at	HNRNPA2B1	0.07032926	0.72984597	Reader
227377_at	IGF2BP1	0.16831684	0.70933601	Reader
218847_at	IGF2BP2	0.3476727	0.42700369	Reader
203820_s_at	IGF2BP3	1.20020404	0.00658052	Reader

**Table 2:** IGF2BP3-RRM2 association.

Reader	Source (cell line/tissues)	GSE ID	Target gene	Peak region
IGF2BP3	K562	GSE138063	RRM2	chr2: 10130913–10130933; chr2: 10127121–10127143; chr2: 10131380–10131410; chr2: 10122859–10122884; chr2: 10129417–10129439
IGF2BP3	REH	GSE76931	RRM2	chr2: 10130639–10130692
IGF2BP3	HEK293T	GSE90639	RRM2	2.4024(Log2FC)
IGF2BP3	RS4;11	GSE76931	RRM2	chr2: 10130630–10130694

**Fig 3:**
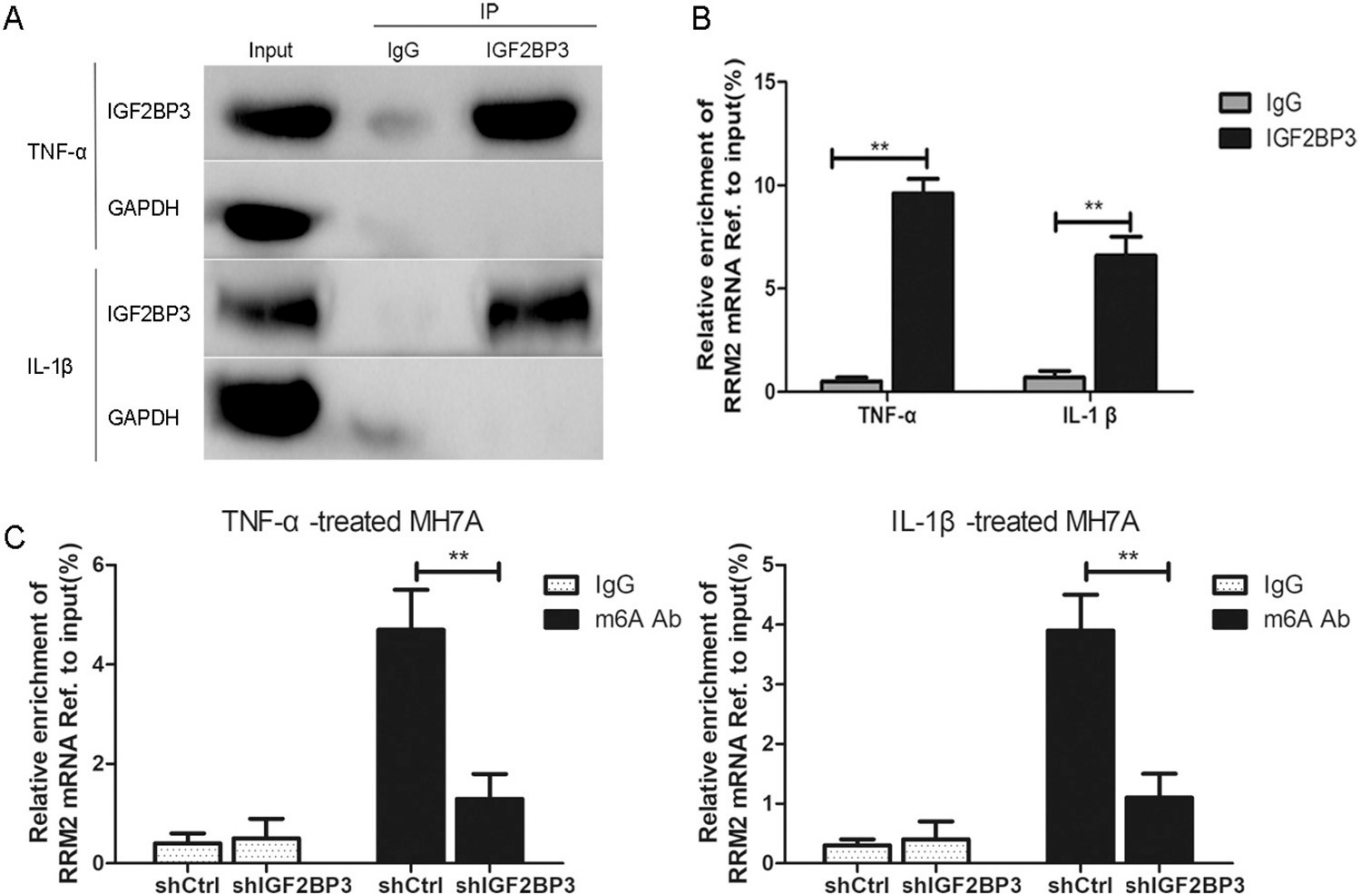
IGF2BP3 increases RRM2 mRNA enrichment via m6A modification. A. IGF2BP3 RIP assay. IGF2BP3 immunoprecipitation was performed and detected by Western blotting in TNF-α or IL-1β-treated MH7A cells. GAPDH and IgG was used as the negative control. B. RIP-qPCR assay demonstrated the enrichment of RRM2 mRNA in in anti-IGF2BP3 precipitates of TNF-α or IL-1β-treated MH7A cells(**p<0.01). C. MeRIP-qPCR assay. The m6A enrichment of RRM2 mRNA was shown by using anti-IgG and anti-m6A antibodies in TNF-α or IL-1β-treated MH7A cells after knocking down IGF2BP3. **P < 0.01.

To validate whether IGF2BP3 interacts with RRM2 mRNA via m6A modification, RIP-qPCR assay was performed using anti-IGF2BP3 antibody in TNF-α or IL-1β-treated MH7A cells. As shown in Fig 3A and 3B, the results showed that RRM2 mRNA was significantly enriched by using IGF2BP3 antibody than that of the IgG controls in TNF-α or IL-1β-treated MH7A cells(**p<0.01), suggesting that IGFBP3 directly interacted with RRM2 mRNA. Furthermore, we performed the meRIP-qPCR assays to test whether RRM2 m RNA was affected by m6A modification. As shown in Fig 3C, knockdown of IGF2BP3 markedly decreased the m6A levels of RRM2 mRNA in TNF-α or IL-1β-treated MH7A cells compared with the negative control shRNA group (**p<0.01).

**Fig 4:**
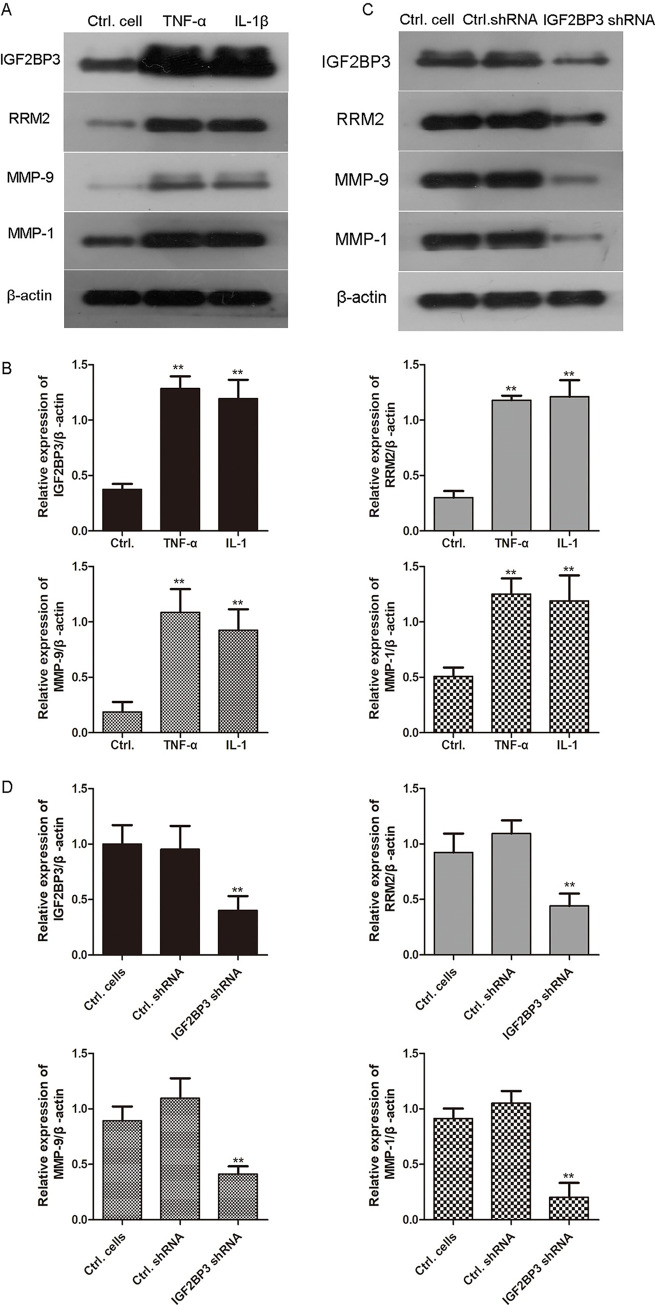
The expression of IGF2BP3 and RRM2 detected via western blotting in MH7A cells. A. MH7A cells were treated with TNF-α (10 ng/mL) and IL-1β (10 mg/L) for 24 h, and the expression of RRM2 was determined via western blotting. B. The relative expression of IGF2BP3, RRM2, MMP-9 and MMP-1 was shown in histogram. **p<0.01, compared with control group. C. MH7A cells were infected with IGF2BP3 shRNA and control shRNA lentiviruses and treated with 10 ng/mL of TNF-α for 24 h. The expression of IGF2BP3, RRM2, MMP-9, and MMP-1 was detected via western blotting. D. The relative expression of IGF2BP3, RRM2, MMP-9 and MMP-1 was shown in histogram. **p<0.01, compared with control shRNA group.

We used TNF-α (10 ng/mL) or IL1-β (10 mg/L) to treat MH7A cells for 24 h to mimic the synoviocytes in patients with RA. As shown in Fig 4, the expression of IGF2BP3 was significantly upregulated in TNF-α or IL1-β- treated cells (**p<0.01). Moreover, the expression of RRM2, MMP-1, and MMP-9 was also significantly increased in TNF-α or IL1-β- treated cells compared with that in control cells (**p<0.01). Consistently, MH7A cells were infected with IGF2BP3 shRNA lentivirus for 48 h, and the expression of IGF2BP3, as well as the expression of RRM2, MMP-1, and MMP-9, was found to be significantly decreased via western blotting.

### IGF2BP3 knockdown inhibits cell proliferation, migration, and invasion of MH7A cells via RRM2

**Fig 5:**
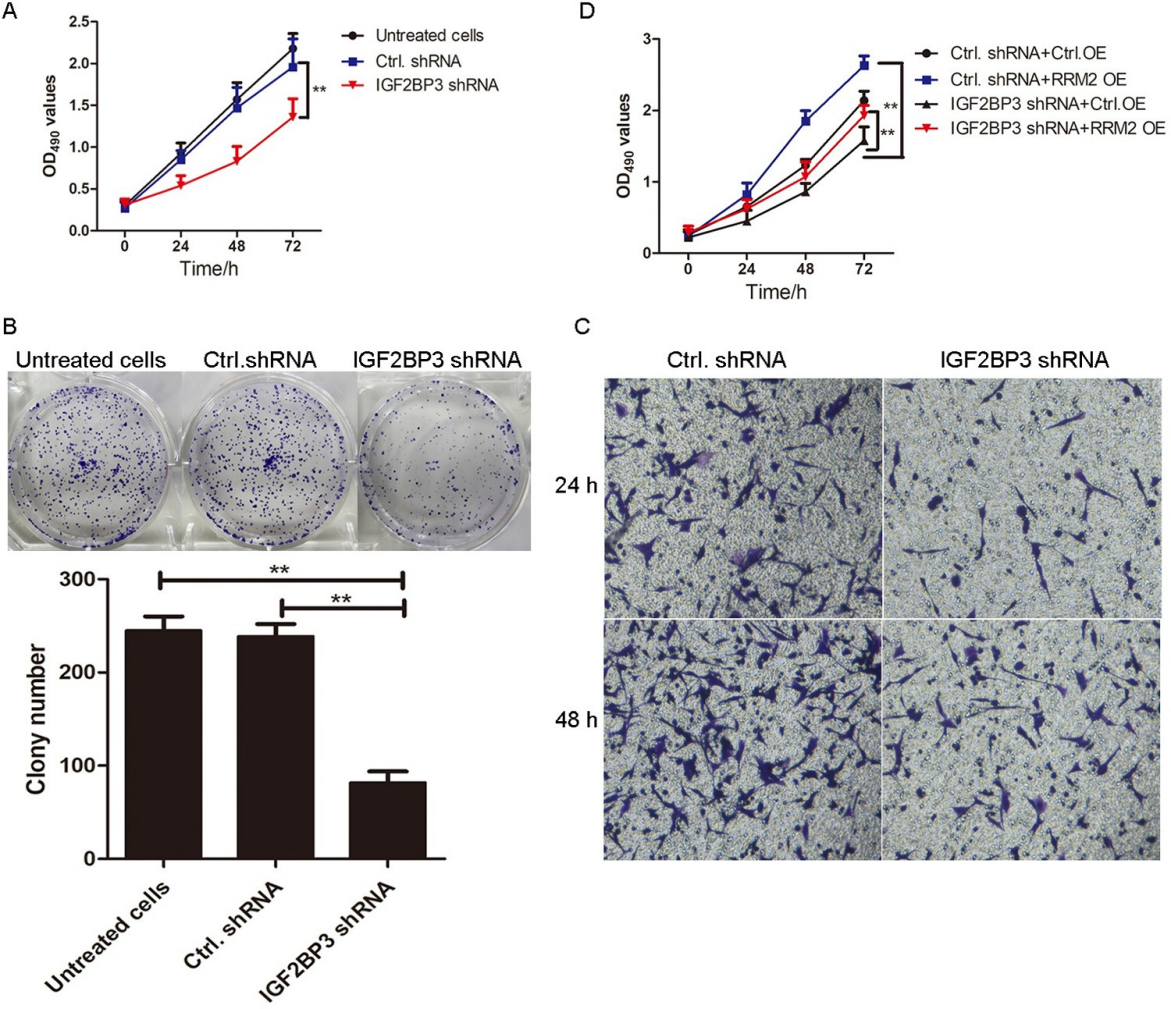
IGF2BP3 knockdown inhibits the proliferation, migration, and invasion of MH7A cells. A. MTT assay. MH7A cells were infected with IGF2BP3 shRNA lentivirus and control shRNA lentivirus was treated with TNF-α (10 ng/mL) for 24, 48, and 72 h. The cell viability was determined using MTT assay. **p<0.01, compared with control shRNA group. B. Clone formation assay. MH7A cells were infected with IGF2BP3 shRNA and control shRNA lentivirus and cultured for 2 weeks. The colony number was shown in histogram. **p<0.01, compared with control shRNA group. C. Migration and invasion assays. The effects of IGF2BP3 knockdown on invasion and migration were detected using transwell Boyden chamber coated with or without a Matrigel basement membrane matrix after 48 h. Original magnification ×100. D. MTT assay. MH7A cells were treated with IGF2BP3 shRNA lentivirus and overexpressed control lentivirus, control shRNA lentivirus and overexpressed RRM2 lentivirus, IGF2BP3 shRNA lentivirus and overexpressed control lentivirus, and IGF2BP3 shRNA lentivirus and overexpressed RRM2 lentivirus for 24, 48, and 72 h. Cell viability was determined using MTT assay. **p<0.01, compared with control group.

MH7A cells were infected with IGF2BP3 shRNA lentivirus and control shRNA lentivirus for 24 h, 48 h, and 72 h, and MTT assay results showed that the cell viability was significantly decreased in IGF2BP3 shRNA lentivirus-infected cells, compared to that in control shRNA lentivirus-infected cells (**p<0.01, Fig 5A). Moreover, the effects of IGF2BP3 knockdown on cell proliferation was determined via colony formation assay. As shown in Fig 5B, the number of cell clones in IGF2BP3 shRNA lentivirus-infected group was significantly less than that in control shRNA lentivirus-infected group (**p<0.01). Furthermore, as shown in Fig 5C, the migratory and invasive ability of IGF2BP3 shRNA lentivirus-infected MH7A cells was significantly decreased compared with that of control shRNA lentivirus-infected cells at 48 h (p<0.01).

To identify whether the effects of IGF2BP3 on proliferation and invasion was dependent on RRM2, we designed the rescue experiment on MH7A cells. As shown in Fig 5D, MH7A cells were infected with IGF2BP3 shRNA lentivirus, with RRM2 overexpressed simultaneously. MTT assay results showed that the inhibitory effects of IGF2BP3 knockdown were effectively reversed by simultaneously overexpressing RRM2 in MH7A cells (**p<0.01). Collectively, the results demonstrated that RRM2 might be the target gene of IGF2BP3 to regulate the proliferation, migration, and invasion of MH7A cells.

### RRM2 regulates the migration and invasion of MH7A cells via RRM2/Akt/MMP-9 pathway

**Fig 6:**
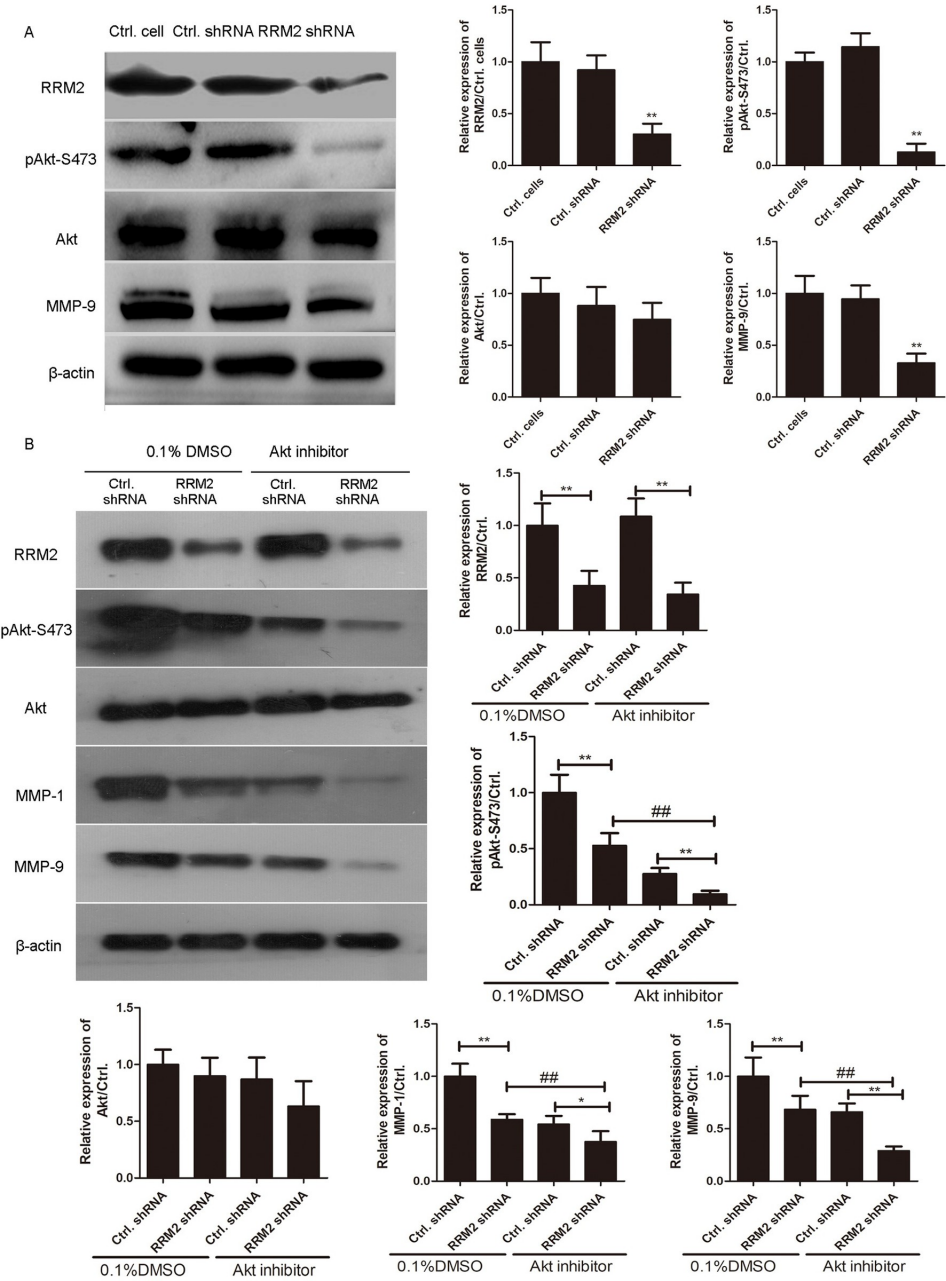
RRM2 regulates the expression of MMP-9 via Akt phosphorylation. A. MH7A cells were infected with RRM2 shRNA or control shRNA lentiviruses for 48 h. The expression of RRM2, phosphorylated Akt, total Akt, and MMP-9 was detected via western blotting. The relative expression of RRM2, p-Akt, Akt and MMP-9 was shown in histogram. **p<0.01, compared with control shRNA group. B. The MH7A cells infected with RRM2 shRNA or control shRNA lentiviruses were treated with Akt inhibitor for 24 h. The expression of pAkt-S473, total Akt, and MMP-9 was detected via western blotting. The relative expression of RRM2, p-Akt, Akt, MMP-1 and MMP-9 was shown in histogram. **p<0.01, ##p<0.01, compared with control group.

To test whether RRM2 regulated the invasion and migration of MH7A cells through Akt-related signaling pathway, we detected the expression of RRM2, phosphorylated Akt, total Akt, and its targeted genes in RA progression via western blotting analysis. First, MH7A cells were infected with RRM2 shRNA or control shRNA lentiviruses for 48 h. As shown in Fig 6, the levels of phosphorylated Akt and MMP-9 were significantly decreased in RRM2 shRNA lentivirus-infected cells, compared with control shRNA lentivirus-infected cells (**p<0.01). Moreover, MH7A cells were infected with RRM2 shRNA or control shRNA lentiviruses in combination with 100 nM Akt selective inhibitor GSK690693 for 24 h. Western blotting analysis results revealed that the phosphorylated Akt-S473 level was significantly decreased in RRM2 shRNA lentivirus-infected MH7A cells treated with 100 nM of GSK690693, compared with that treated with 0.1% dimethylsulfoxide (DMSO) for 24 h (**p<0.01). Importantly, the expression of target genes MMP-1 and MMP-9 was significantly decreased in RRM2 shRNA lentivirus-infected MH7A cells in combination with 100 nM of GSK690693, compared with that treated with 0.1% DMSO for 24 h (**p<0.01). The data revealed that RRM2 inhibited cell invasion and migration through RRM2/Akt/MMP9 signaling pathway in MH7A cells.

## Discussion

RA is a common synovial disease with cartilage and bone destruction characterized by leucocyte invasion and synoviocyte activation [29, 30]. Chronic tissue inflammation was found in patients with RA. By analyzing the overlapping DEGs associated with RA in datasets GSE55235, GSE55457 and GSE1919, PPI network was analyzed by using STING database, and the top 10 hub genes identified using Cytoscape and ranked using the MCC[10]. In the present study, we evaluated the expression of 10 hub genes in GSE77298 datasets; an upregulation of RRM2 was identified and further detected in clinical specimens of patients with RA. We also used TNF-α and IL1-β to treat MH7A cells to construct a cell model of RA and studied the molecular mechanism of RRM2 in the progression of RA. Consistently, the expression of RRM2 was significantly increased in TNF-α and IL-1β-treated MH7A cells. Other researchers have found that RRM2 was highly expressed in PBMCs collected from patients with RA compared to healthy controls [11], consistent with our earlier results [10]. Moreover, RRM2 showed a high diagnostic value for patients with RA with area under curve = 0.941 (p<0.0001; sensitivity = 0.867) [11]. The results suggest that RRM2 overexpression may contribute to synoviocyte survival and synovial hyperplasia during RA progression. As RRM2 was highly expressed in RA progression, it could be used as an effective therapeutic target for patients with RA.

Actually, the relationship between cancer and RRM2 was widely reported. For example, RRM2 was thought as an independent predictive factor of poor prognosis of LUAD [31] and breast cancer [9]. RRM2 protected against ferroptosis and worked as a tumor biomarker in liver cancer [32]. It was reported that RRM2 alleviated chemotherapeutic drug resistance through the Akt/mTOR signaling [33]. But till now, few studies were found on m6A modification of RRM2 in the progression of RA. In the present study, we found that the m6A reader IGF2BP3 and RRM2 were highly expressed in clinical specimens and TNF-α and IL-1β-stimulated synovial cells. By analyzing m6A2Target database, 5 binding sites in RRM2 mRNA were found, which was the possible m6A regulatory target for IGF2BP3, suggesting a direct relationship between IGF2BP3 and RRM2 mRNA. RRM2 and IGF2BP3 knockdown inhibited the proliferation, migration and invasion of MH7A cells. Interestingly, the rescue experiment results showed that the inhibitory effects of IGF2BP3 knockdown were effectively reversed by over-expressing RRM2 simultaneously in MH7A cells. Moreover, RRM2 regulated the migration and invasion of MH7A cells via RRM2/Akt/MMP-9 pathway to promote the progression of RA. Thus, in the present study, we elucidated the mechanisms whether and how RRM2 was regulated by IGF2BP3 probably in a m6A-dependent manner in RA progression. The m6A modification plays an important role in RA pathogenesis. We first analyzed the expression of several key m6A regulatory genes in GSE77298, and the results demonstrated that IGF2BP3 was upregulated in patients with RA, which was further confirmed in TNF-α and IL-1β-treated MH7A cells. The m6A2Target database (http://m6a2target.canceromics.org) is a comprehensive database for targets of m6A writers, erasers, and readers. Here, we analyzed the m6A regulators, including writers, erasers, and readers, and found that IGF2BP3 was one of the possible m6A readers of RRM2 with five binding sites in RRM2 mRNA. Interestingly, the inhibition was reversed in IGF2BP3 knockdown MH7A cells simultaneously overexpressing RRM2. Taken together, these results suggest that IGF2BP3 might stabilize and promote the expression of RRM2 in an m6A-dependent manner, which should be further identified and clarified in near future. Next, the involvement of m6A-dependent regulators, including m6A writers, erasers, and readers, in the progression of RA should be further be verified in near future. Additionally, more clinical specimens should be collected and used to test the expression of IGF2BP3 and RRM2 in patients with RA and controls. Moreover, we need design more experiments to investigate and prove the question: whether the regulation of RRM2 and N6-methyladenosine are specific in RA. For instance, the m6A modification experiment was need to tested and validated in the other disease models, such as cancer cell lines. This is interesting, but we need more time and experiments to resolve and answer it in future.

Next, we wanted to clarify the related signaling pathway with RRM2 in progression of RA. It has been found that miR-1 inhibited the proliferation and metastasis of small cell lung cancer via C-X-C chemokine receptor type 4 / forkhead box protein / RRM2 axis [34]. Moreover, miR-20a-5p inhibited tumor angiogenesis of NSCLC through RRM2/PI3K/Akt signaling pathway [18]. Additionally, RRM2 regulated the invasiveness of gastric cancer cells through Akt/NF-kB signaling pathway [35]. It has been reported that Akt expression was significantly high in the synovial tissues of RA patient [36], consistent with the results of our study. In the present study, we found that RRM2 knockdown significantly suppressed the expression of MMP-1 and MMP-9 in TNF-α-treated MH7A cells, which was mediated by the phosphorylation of Akt. The results suggest that RRM2 promoted Akt phosphorylation, leading to high expression of MMP-1 and MMP-9 to enhance the migration and invasive capacities of MH7A cells. The data revealed that IGF2BP3-mediated RRM2 promoted pathogenesis of RA via the phosphorylation of Akt and upregulated the expression of MMP-1 and MMP-9 to promote the migration and invasion of MH7A cells.

In conclusion, we found that RRM2 regulated the migration and invasion of MH7A cells via RRM2/Akt/MMP-1, -9 pathway, which was regulated by m6A reader IGF2BP3, possibly in an m6A-dependent manner. This reveals an IGF2BP3-related m6A modification of RRM2 as an effective therapeutic target in patients with RA. Thus, it would be helpful and meaningful to explore RRM2-specific inhibitors more effectively to obtain additional potential therapeutic implications in patients with RA.

## Supporting information

S1 Table (XLSX)

S1 File (XLSX)

S2 File (DOCX)
