# Supplementary material for: IGF2BP3 regulates the expression of RRM2 and promotes the progression of rheumatoid arthritis via RRM2/Akt/MMP-9 pathway
Source: PLoS One. 2024 May 31;19(5):e0303593. doi: 10.1371/journal.pone.0303593 (PMC11142689; doi:10.1371/journal.pone.0303593)
Supplement: S2 File — (DOCX) [file pone.0303593.s003.docx]

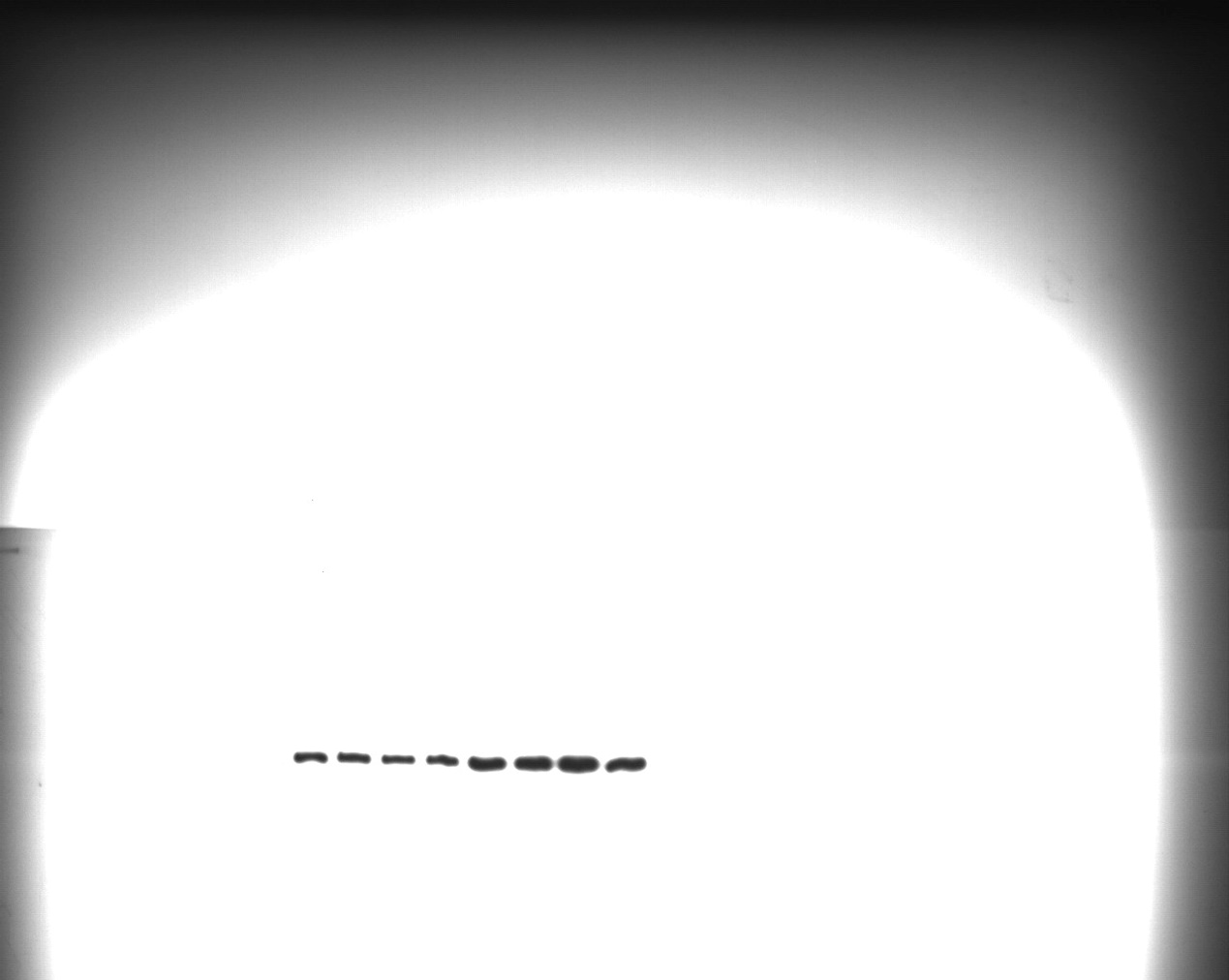


Figure 2A RRM2


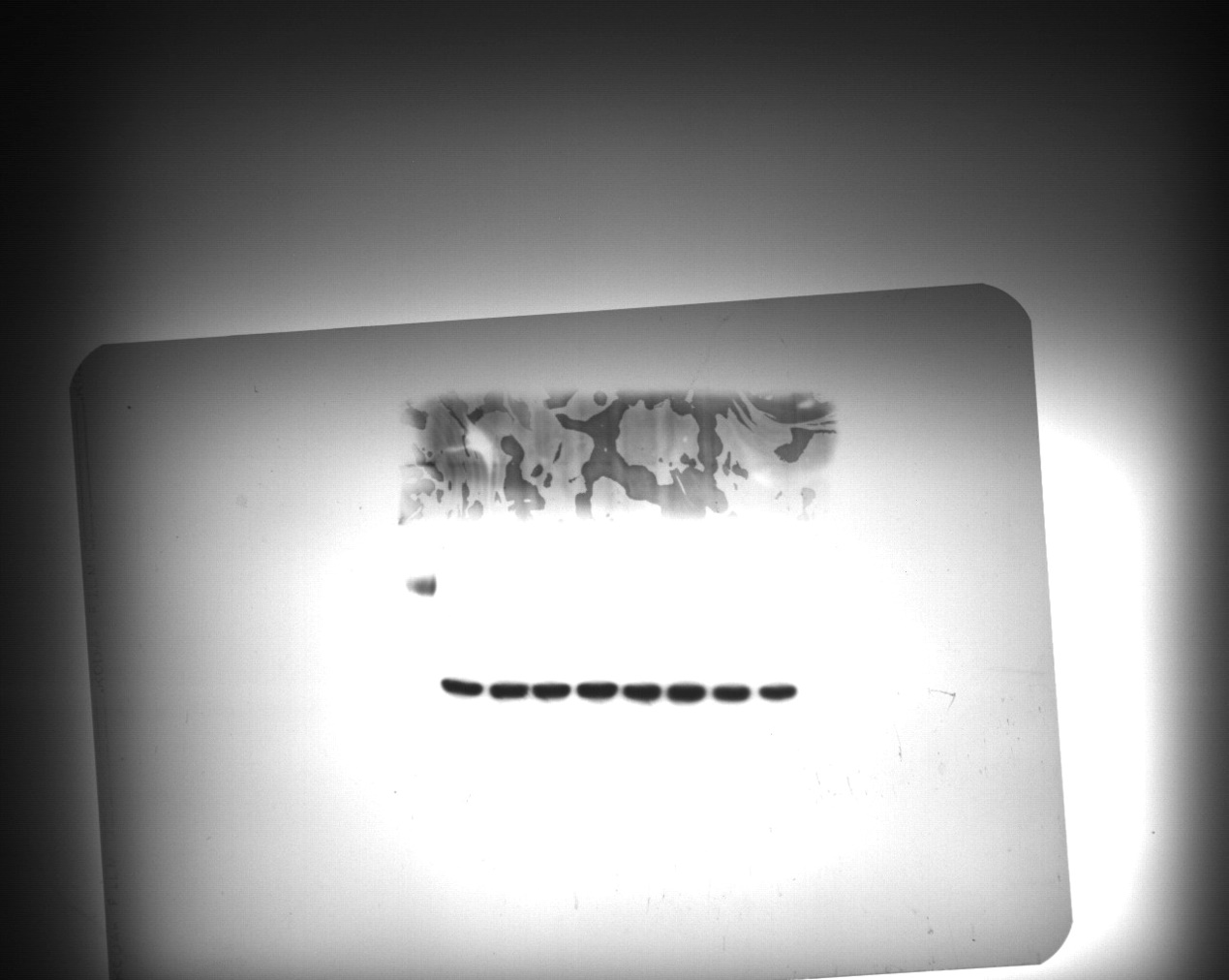


Figure 2A beta-actin


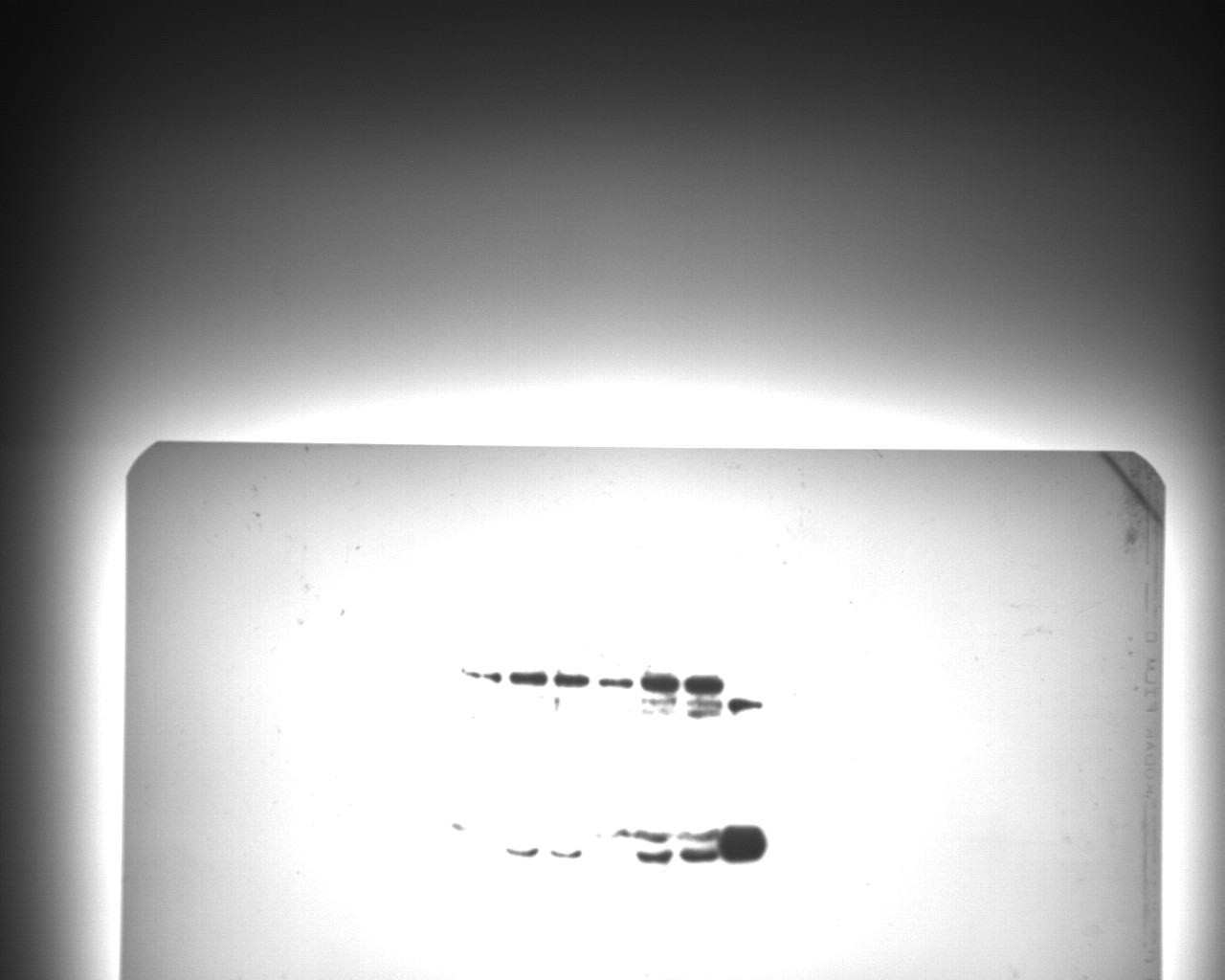


Figure 2B RRM2


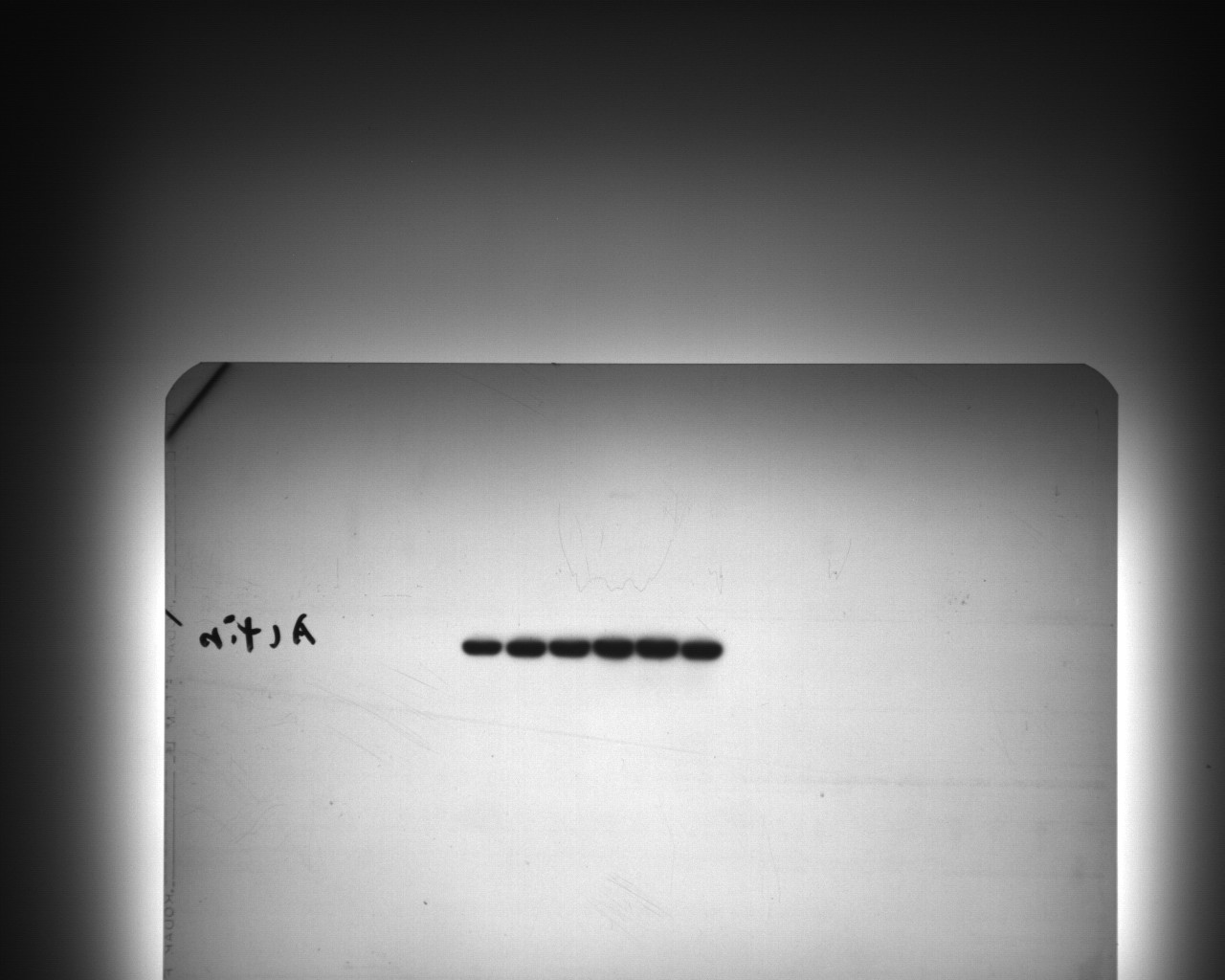


Figure 2b beta-actin


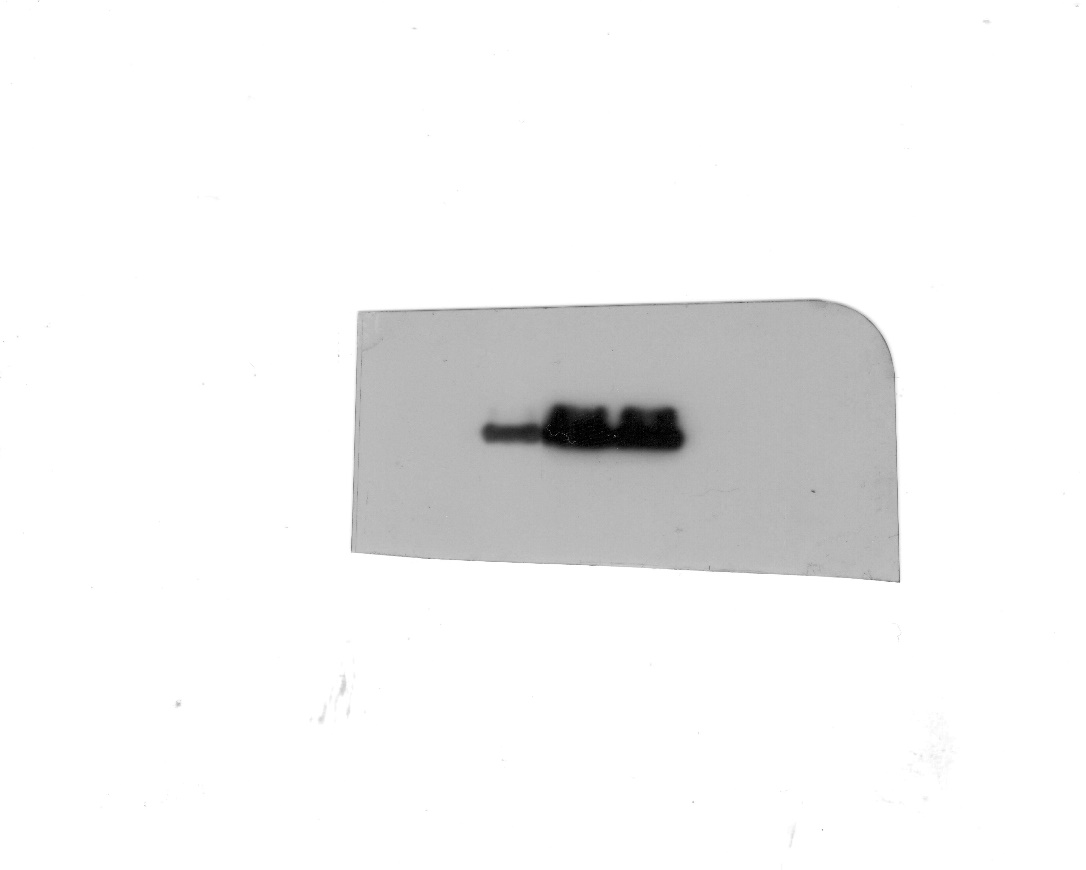


Figure 4 IGF2BP3


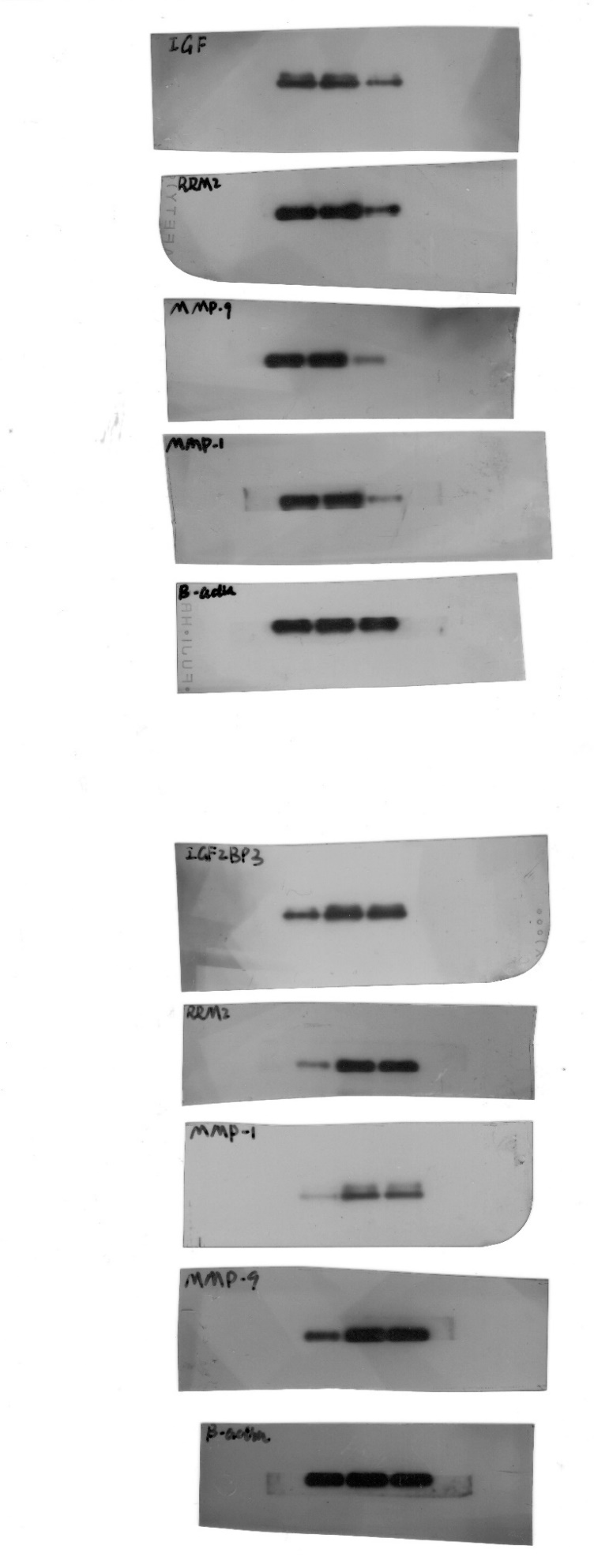


Figure 4.


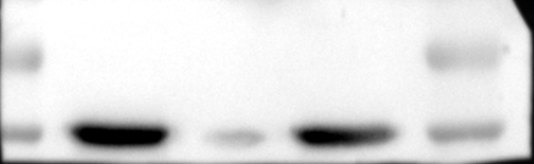

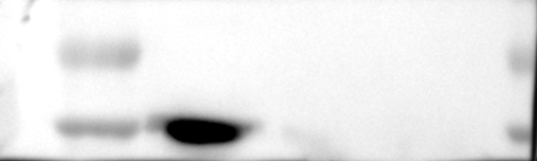


IGF2BP3

70KD

40KD

GAPDH


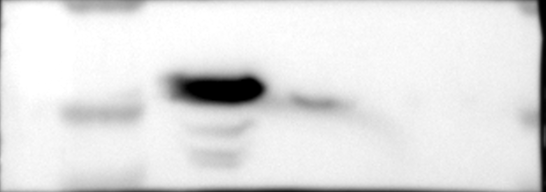

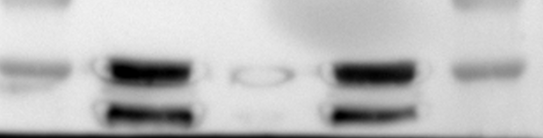


IGF2BP3

70KD

40KD

GAPDH

Figure 3


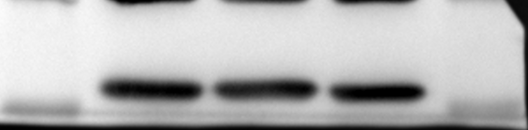

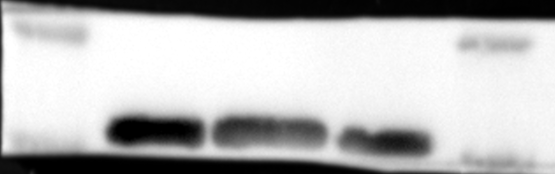

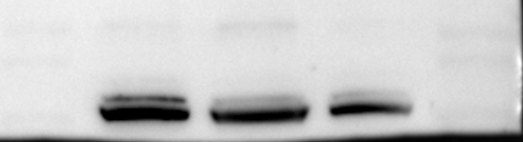

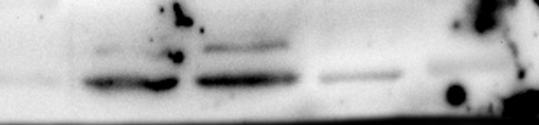


100KD

70KD

35KD

40KD

RRM2

β-actin

p-AKT

MMP9


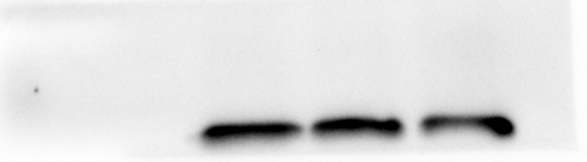

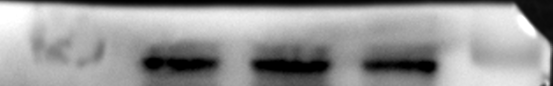


AKT

70KD

40KD

Β-actin

Figure 6A The second time for the experiment.


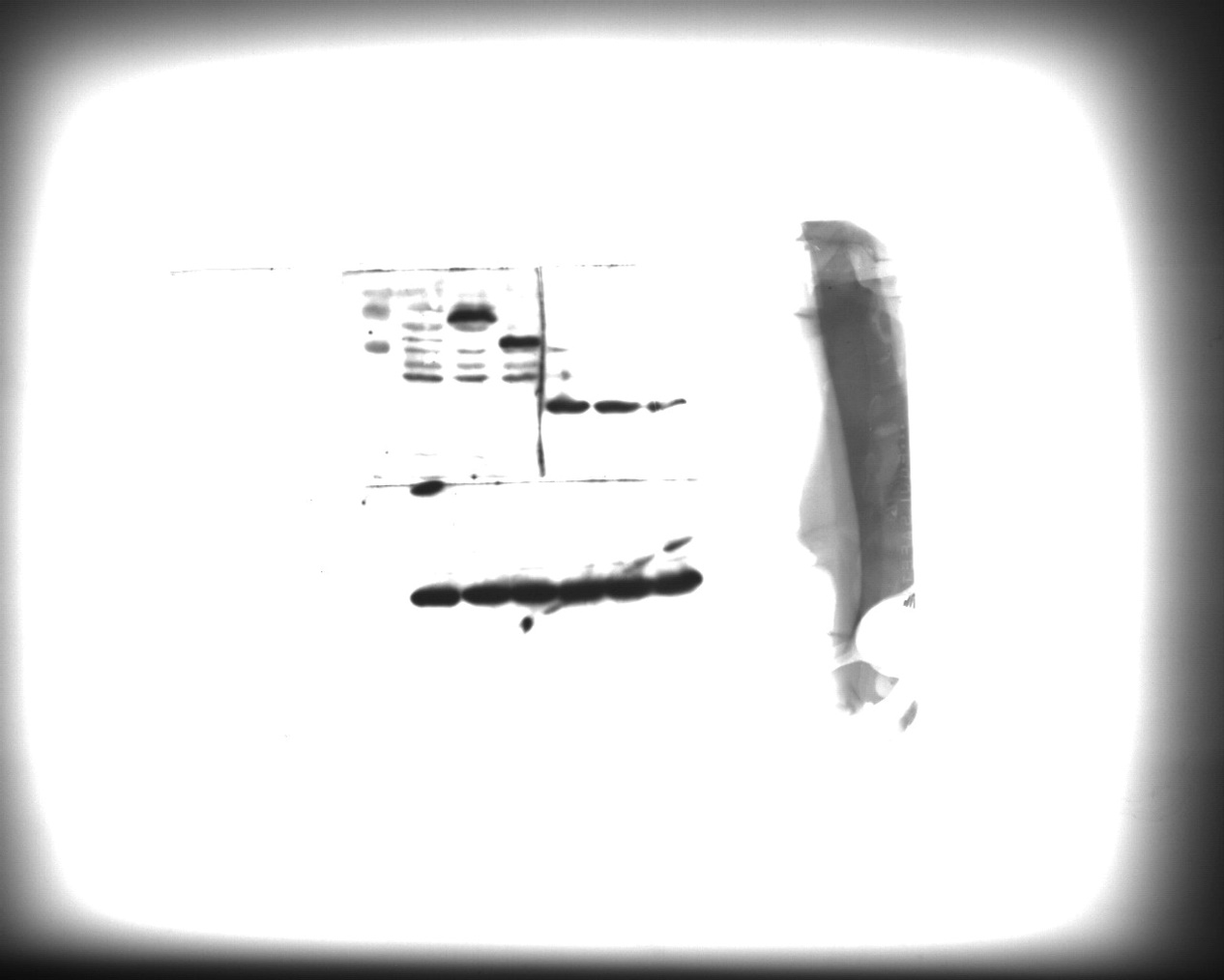


Figure 6 RRM2 first time


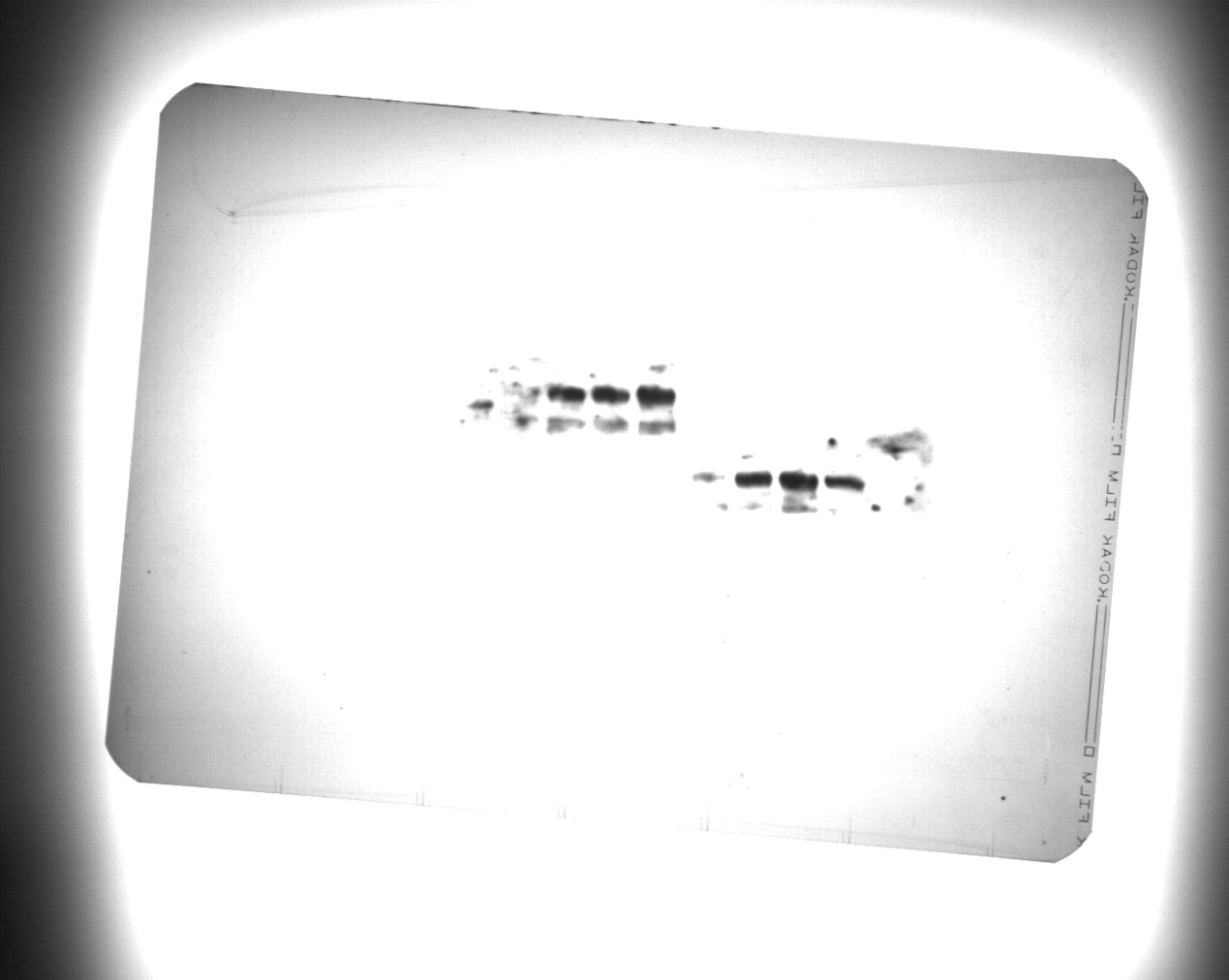


Figure 6 MMP9-first time


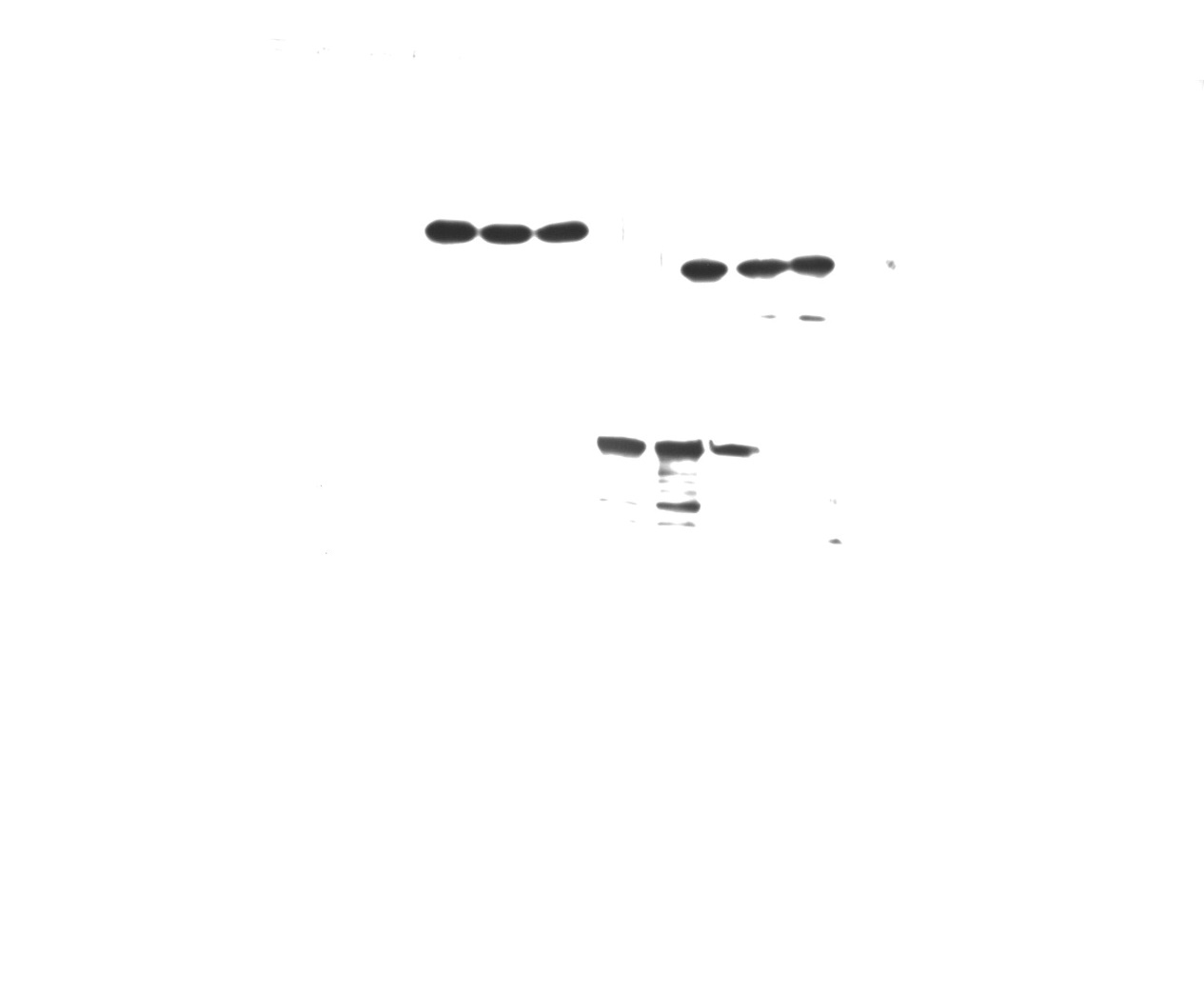


Figure 6 pAkt Akt and beta-actin -first time


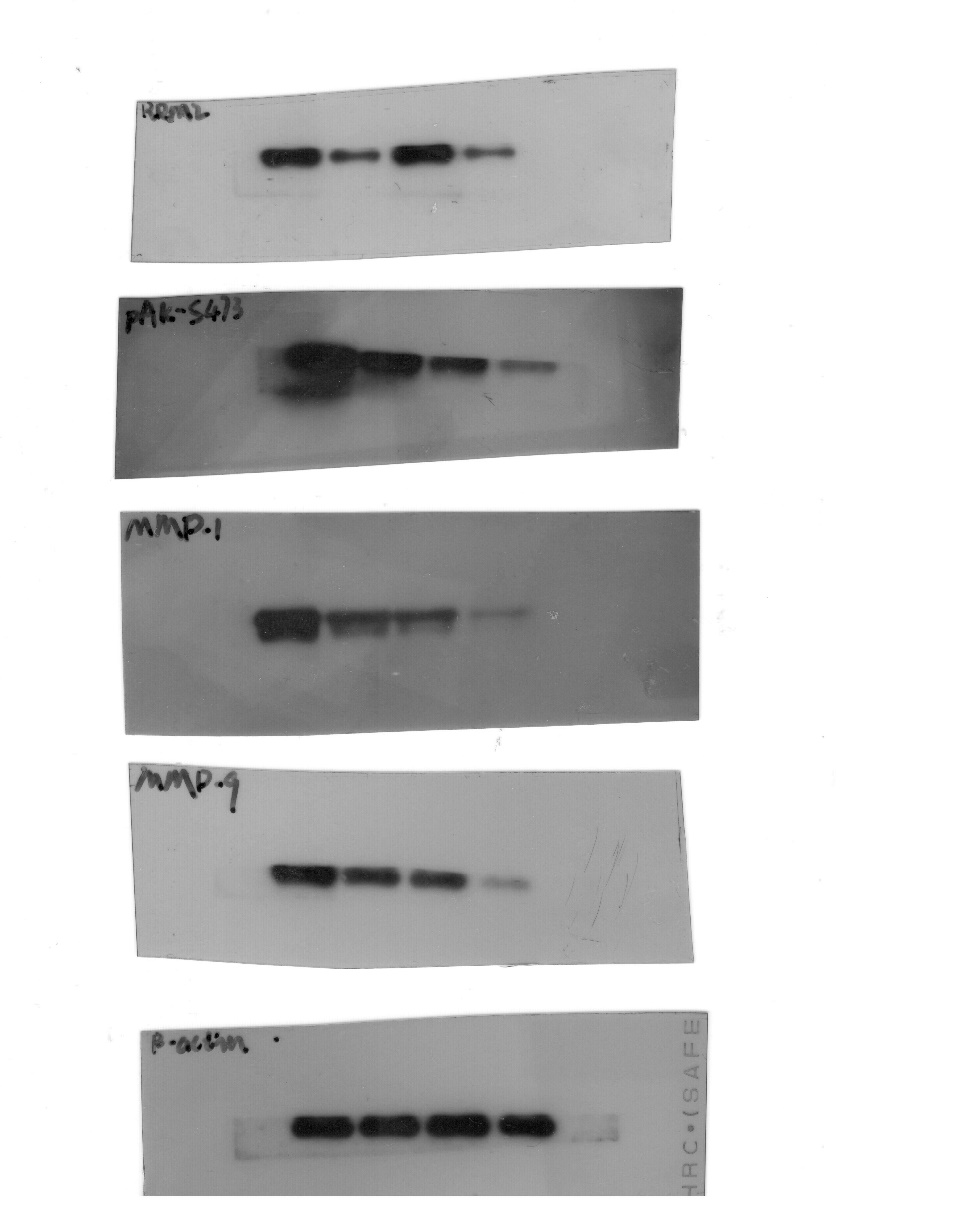


Figure 6B
